# Positive–negative-selection-mediated gene targeting in rice

**DOI:** 10.3389/fpls.2014.00748

**Published:** 2015-01-05

**Authors:** Zenpei Shimatani, Ayako Nishizawa-Yokoi, Masaki Endo, Seiichi Toki, Rie Terada

**Affiliations:** ^1^Laboratory of Plant Molecular Genetics, Graduate School of Biological Sciences, Nara Institute of Science and TechnologyIkoma, Japan; ^2^Plant Genome Engineering Research Unit, Agrogenomics Research Center, National Institute of Agrobiological SciencesTsukuba, Japan; ^3^Development of Agrobiological Resources, Faculty of Agriculture, Meijo UniversityNagoya, Japan

**Keywords:** gene targeting, homologous recombination, positive–negative selection, rice, knock-in, marker-free, site specific recombination, gene editing

## Abstract

Gene targeting (GT) refers to the designed modification of genomic sequence(s) through homologous recombination (HR). GT is a powerful tool both for the study of gene function and for molecular breeding. However, in transformation of higher plants, non-homologous end joining (NHEJ) occurs overwhelmingly in somatic cells, masking HR-mediated GT. Positive–negative selection (PNS) is an approach for finding HR-mediated GT events because it can eliminate NHEJ effectively by expression of a negative-selection marker gene. In rice—a major crop worldwide—reproducible PNS-mediated GT of endogenous genes has now been successfully achieved. The procedure is based on strong PNS using *diphtheria toxin A-fragment* as a negative marker, and has succeeded in the directed modification of several endogenous rice genes in various ways. In addition to gene knock-outs and knock-ins, a nucleotide substitution in a target gene was also achieved recently. This review presents a summary of the development of the rice PNS system, highlighting its advantages. Different types of gene modification and gene editing aimed at developing new plant breeding technology based on PNS are discussed.

## ADVANTAGES OF DEVELOPING A PNS SYSTEM IN RICE

In higher plants, the establishment of GT of endogenous natural genes through HR has been hampered by the overwhelming occurrence of NHEJ, i.e., random recombination, even when the transformed gene carries sequence(s) homologous to the target gene locus. Despite the clear demonstration of GT at an artificially generated selectable locus in tobacco somatic cells (Paszkowski et al., 1988), the frequency of GT was estimated to be 10^-3^ to 10^-6^ that of random integration. To overcome the low frequency of HR, various approaches for enhancement of HR and/or reduction of NHEJ have been attempted based on our existing knowledge of genome recombination and repair (Britt and May, 2003). In *Arabidopsis*, the yeast *RAD54* gene—a member of the *SWI2*/*SNF2* chromatin remodeling gene family—enhances GT frequency (Shaked et al., 2005); however, the procedure was still not efficient enough to detect GT of various endogenous genes. Induction of a DSB at the target site using an artificial endonuclease is now progressing as a means of establishing GT in several plant species (Shukla et al., 2009; Zhang et al., 2013; Endo and Toki, 2014; Puchta and Fauser, 2014), although most DSBs re-connected by NHEJ result in target gene disruption.

**FIGURE 1:**
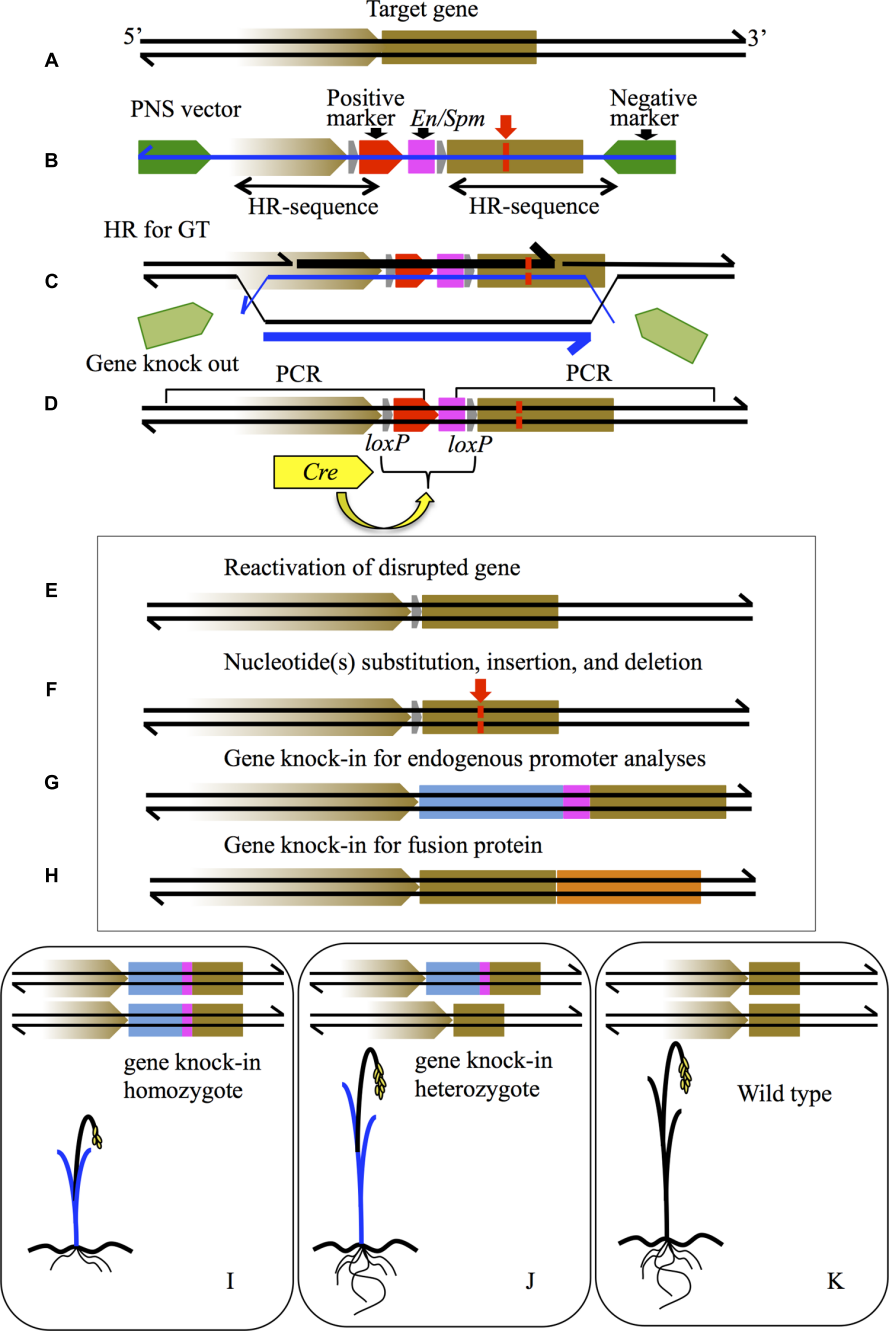
**Schematic diagram of various gene modifications by PNS-mediated GT. (A)** The brown box indicates the gene to be targeted on a genome sequence shown as black lines. The brown arrow represents the promoter of the gene. **(B)** PNS vector for GT. The green arrows are the negative markers; the red arrow is the positive marker. The pink box is the transcriptional stop sequence of *En/Spm*. The gray arrows are *loxP* sequences. Double-headed arrows under the vector indicate the homology regions for HR. The blue line is T-DNA sequence. **(C)** HR process for GT between the target gene and PNS vector. The thick lines of black and blue indicate newly synthesized DNA sequences in genome and T-DNA, respectively. **(D)** Gene knock-out of the target gene by insertion of a positive marker with *En/Spm*, which can be removed via subsequent Cre-*loxP* recombination caused by introduced *Cre* gene (yellow arrow). **(E)** Reactivation of knock-out gene in **(D)** by Cre-*loxP* recombination. **(F)** Nucleotide(s) substitution (red lines), insertion, and deletion in the target gene can be induced by designing a homology arm in the PNS vector in **(B)** and subsequent positive marker elimination by Cre-*loxP* recombination in **(D)**. **(G)** Gene knock-in modification where the endogenous promoter sequence is connected to the *GUS* coding sequence (indicated as a blue box with *En/Spm*). **(H)** Gene knock-in modification where the *mOrange* coding sequence, indicated as an orange box, is connected precisely to the stop codon of the target gene; both endogenous promoter activity and protein localization of the target gene are detectable. **(I–K)** Diagrams of segregated plants from knock-in T_0_ into homozygote **(I)**, heterozygote **(J)**, and wild type **(K)**. GUS expression image as blue leaves is shown in **(I,J)**. Dwarf phenotype in **(I)** is a reflection of the disrupted target gene.

Positive–negative selection is a strategy for enriching transgenic cells carrying a targeted gene replacing an endogenous gene from among a large number of NHEJ-mediated random recombinants. PNS was first developed for gene knockouts in mice (Mansour et al., 1988). In the higher plant rice (*Oryza sativa* L.)—an important staple food crop—a reproducible PNS-mediated GT procedure applicable to endogenous genes was developed by Terada et al. (2002). In this latter study, the single copy *Waxy* locus (Os06g0133000) was targeted for knockout using a PNS vector carrying the *hpt* gene for positive selection followed by the effective transcriptional stop signal of the maize transposon *En/Spm*, positioned between the *Waxy* homologous sequences; two negative selection genes of *DT-A* (*diphtheria toxin A-fragment* from *Corynebacterium diphtheriae*) flanked both ends of the homologous sequence (**Figures 1A,B**). The *DT-A* acts as a counter-selection agent against NHEJ-mediated random and non-targeted recombinants, and is itself removed by HR between the target locus and the PNS vector (**Figure 1C**). DT-A induces ADP-ribosylation of elongation factor 2 in eukaryotic ribosomes and thus prevents protein synthesis (Pappenheimer, 1977; Iida and Terada, 2005). Because DT-A lacks the migration function, the negative selection is cell specific without any effect on neighboring cells (Day and Irish, 1997; Iida and Terada, 2004, 2005). To ensure strong selection against a large number of background recombinants, highly active promoters from the rice *Actin1* gene (including its intron), cauliflower mosaic virus (*CaMV35S* with *the caster bean catalase* intron), and the maize *Ubiquitin* gene (also with its intron) were employed to express PNS markers in large-scale T-DNA-mediated rice transformation experiments (Terada et al., 2002, 2004). GT via HR was identified by PCR analysis of calli surviving PNS by detection of targeted-specific sequences reflecting insertion of the *hpt*-*En/Spm* into the *Waxy* locus (**Figure 1D**). Most survivors of PNS were derived from the random integration of the GT vector in which the *DT-A* genes have become non-functional due to rearrangements of the sequences (Terada et al., 2007). The GT frequency was calculated as 6.4 × 10^-4^ based on total transformants (six targeted lines per 9,300 calli), which lies within the range of 10^-3^ to 10^-6^ predicted in earlier GT experiments with an artificially generated selectable target gene locus (Paszkowski et al., 1988). We generally use the percentage of targeted lines obtained per number of surviving calli on PNS to define the efficiency of GT, in our case 0.94 % (six targeted lines per 638 calli). The heterozygosity of the *Waxy* locus in targeted T_0_ plants was confirmed by Southern blot and DNA sequence analysis at the *waxy* locus and by the Mendelian segregation of the *Waxy-waxy* phenotype in T_1_ plants (Terada et al., 2002).

Because PNS-mediated GT occurs via HR between homologous sequences present on both the vector and a corresponding sequence at the targeted locus (**Figures 1B–D**), the procedure could be used to introduce desired mutations of various types into any gene of interest (**Figures 1E–H**). After the first successful GT of the *Waxy* locus (Terada et al., 2002), many endogenous rice genes (more than 10 loci) have been targeted and altered to desired forms (Terada et al., 2007; Yamauchi et al., 2009, 2014; Moritoh et al., 2012; Ono et al., 2012; Ozawa et al., 2012; Dang et al., 2013; Osakabe et al., 2014). At the early stage of PNS development for higher plants, the *codA* gene was employed for negative selection rather than *DT-A* because the toxic effect of DT-A was not only non-conditional but also very strong, so that even transient expression of *DT-A* would kill any cell receiving the PNS vector. On the other hand, the toxic effect of DT-A expressed transiently can be suppressed by the following T-DNA-mediated transformation process, compared with results obtained in direct transformation methods delivering a double-stranded DNA vector in GT experiments in mice and yeast. The applicability of T-DNA for HR-mediated GT has been confirmed for an artificially generated selectable target locus in tobacco (Offringa and Hooykaas, 1995). T-DNA-mediated transformation of the monocot plant rice—a non-host of *Agrobacterium*—was developed by Hiei et al. (1994). A single-stranded T-DNA carrying the GT vector for PNS is transformed into the plant nucleus, where it is then thought to be converted into double-stranded DNA (dsDNA), and integrated into the host genome (Loyter et al., 2005). Transient expression of *DT-A* could be delayed due to the time required for ssDNA to dsDNA conversion of T-DNA.

The *codA* gene, which encodes cytosine deaminase [catalyzes conversion of 5-fluorocytosine (5-FC) to the toxic 5-fluorouracil (5-FU)], was used as a conditional negative marker for establishment of PNS-mediated GT in *Lotus japonicas* and *Arabidopsis thaliana*; however, *codA* was found to be insufficient for negative selection of GT events (Thykjaer et al., 1997; Gallego et al., 1999; Wang et al., 2001; Iida and Terada, 2005). The *codA* gene can be improved by introducing a single amino acid substitution: D314A (Mahan et al., 2004), and negative selection using this modified *codA* (D314A) was recently found to be functionally comparable to that using *DT-A* (Osakabe et al., 2014). The rice *CAOMT* (caffeic acid *O*-methyltransferase) gene was targeted successfully by PNS using modified *codA* (D314A). Development of suitable negative selection markers is important to improve PNS-mediated GT and to make it more publicly acceptable, especially as a procedure for molecular breeding. The embryonic rice calli used for PNS-mediated GT, which maintain totipotency for regeneration, are postulated to be as HR-reactive as mouse embryonic stem cells (where HR is common, occurring with a frequency of more than 10^-2^ among transformation events; Jasin et al., 1996). Such calli consist of small, compact, and vigorously proliferating cells that have the additional advantage of being easy to handle for large scale-transformation (Terada et al., 2004).

## APPLICATIONS OF PNS AND THE VARIETY OF POSSIBLE GENE MODIFICATIONS

As shown in **Figures 1D–H**, genome sequences can be modified to various forms by PNS-mediated GT, i.e., not only gene knockouts but also gene knock-ins have been established, as well as nucleotide insertions, deletions, and substitutions. In addition to the *Waxy* knockout, the *Alcohol dehydrogenase2* (*Adh2*) gene, Os11g0210500 (Terada et al., 2007) and *Adh1* (Os11g0210300) on chromosome 11 were targeted independently by the same PNS strategy, despite both genes being surrounded by redundant sequences of repetitive Copia-like and Gypsy-like retroelements (Tarchini et al., 2000; Iida and Terada, 2005). Recently, it was shown that disruption of the single copy rice gene *Xyl*, encoding β1,2-gxylosyl-transferase, resulted in the absence of xylose residues in targeted homozygotes (Ozawa et al., 2012). To date, more than ten gene loci distributed in different positions on rice chromosomes have been targeted and altered to different forms. GT efficiency ranges from about 1.0–10 % among PNS survivors, and is assumed to depend on characteristics of the DNA sequence required for HR, such as sequence repeat(s) and palindromic elements, as well as other genomic processes such as DNA replication and/or transcription, and epigenetic modifications of DNA and chromosome(s).

In general, gene promoter activities can be studied by analyzing transgenic plants carrying chimeric genes with the promoter of interest fused to the coding sequence of a visual marker such as *GUS* or *GFP,* although expression of visual markers can be unstable depending on positional effects and multicopy integrations of the chimeric gene (Yamauchi et al., 2009). In addition, promoters in chimeric genes do not always reflect their original functionality because of the length limitation of promoter regions that can be applied for gene transformation. In a knock-in GT experiment, the *GUS* coding sequence attached to *hpt*-*En/Spm* was connected to the promoter of the target gene (**Figure 1G**; Yamauchi et al., 2009, 2014). Because almost all PNS-mediated GT in rice occurs in a heterozygous manner without any additional insertion of the GT vector (Terada et al., 2002, 2007), endogenous promoter activity in the original gene locus can be detected in the targeted heterozygote (**Figure 1J**). Simultaneously, the phenotypic alteration derived from the targeted gene function in addition to endogenous promoter activity is detectable in the targeted homozygote (**Figure 1I**) when compared to the segregated wild type homozygote as a control plant (**Figure 1K**).

Genes functioning in genome methylation were studied by knock-in (plus simultaneous knock-out) GT experiments. The genes for maintenance of CG methylation, *methyltransferase OsMet1a*, Os03g0798300 (Yamauchi et al., 2009) and *OsMet1b*, Os07g0182900 (Yamauchi et al., 2014) were selected based on DNA sequence characteristics and encoded protein motifs, and then targeted precisely. A functional ATG in the target gene was detected by 5′ RACE analyzes and adjusted to become the initiation codon of *GUS* in the knock-in vector. Strong GUS expression was detected in tissues with active cell division, such as meristems in shoot and root, in addition to callus tissue in knock-in plants of *OsMet1a* and *OsMet1b,* respectively. Due to the knock-in of this single locus, dose-dependent GUS expression reflecting targeted-homozygote and -heterozygote was detected clearly in a *OsMet1a* knock-in mutant (Yamauchi et al., 2009). In addition, promoter activities of *OsMet1a* and *OsMet1b* were detected as GUS expression in shoot and root in knock-in hetero- or homozygotes. The original promoter activity of *OsMet1a* and *OsMet1b* was precisely compared through GUS expression in shoots of GT- derived heterozygotes (Yamauchi et al., 2014).

*Domains rearranged methylase 2*, *OsDRM2*, Os03g0110800 (Moritoh et al., 2012), encoding both *de novo* and non-CG *methyltransferase*, and *Repressor of Silencing*, *OsROS1a*, Os01g11900 encoding DNA *demethylase* (Ono et al., 2012) were altered by a knock-in approach by PNS-mediated GT. Whereas no morphological phenotype was detected in *Arabidopsis drm1 drm2* mutants, in rice, the *OsDRM2* knock-in homozygote exhibited drastic growth delay, dwarfism, and sterility, indicating the unique function of *OsDRM2* (Moritoh et al., 2012). *Osros1a*-*GUS* was detected in pollen and unfertilized ovules; concomitantly, an arrest of endosperm growth was observed in heterozygous knock-in rice (Ono et al., 2012). All these results show that the PNS-mediated GT procedure is able to generate novel mutant rice plants based on information gleaned from DNA sequence(s) and encoded protein motif(s), and that the functions of both endogenous promoters and genes can be studied effectively with plants segregated for the targeted locus.

Recently, visual markers such as *GFP*, *mOrange*, and *AsRed2* have been developed, expression of which is detectable in living plant tissues without chemical treatment(s). Connecting sequences encoding these visual markers to the 3′-end of the target gene results in expression of chimeric fusion proteins that allow the spatiotemporal localization of the protein of interest to be visualized (**Figure 1H**).

## POSITIVE-MARKER FREE GENE EDITING BY PNS-MEDIATED GT INDUCED BY SITE-SPECIFIC RECOMBINATION

Positive–negative selection-mediated GT can be used to introduce nucleotide substitution(s) at a targeted locus. Indeed, several nucleotides of the *Adh2* locus have been substituted successfully by a modified GT vector through HR (Johzuka-Hisatomi et al., 2008; **Figure 1F**). Furthermore, because the positive marker of the *hpt*-*En/Spm* is placed between the two *loxP*s in the same orientation, the positive marker can be removed by Cre-*loxP*-mediated site-specific recombination after GT (**Figures 1D–F**). In *Waxy* GT, the *hpt*-*En/Spm* between two *loxP*s was indeed eliminated by transient expression of the *Cre* gene, which was transformed into calli derived from the targeted-*waxy* homozygote, resulting in *Waxy* reactivation in pollen (**Figure 1E**; Terada et al., 2010). These results indicate that nucleotide(s) in rice genome sequence can be substituted precisely by PNS-mediated GT followed by Cre-*loxP* recombination to excise the positive marker.

The *OsRac1* gene (Os01g0229400) was edited by introducing a single nucleotide substitution of G56T, which results in a constitutively active enzyme, by GT-mediated single nucleotide substitution and subsequent positive marker elimination. OsRac1 belongs to the Rac/Rop small GTPase family and acts as a molecular switch in rice immunity. The substitution of guanine (G) with thymine (T) at the 56th nucleotide in exon1 of *OsRac1* alters the 19th glycine (G) to valine (V). The mutated OsRac1(G19V) is constitutively active and increases resistance to rice blast fungus (*Magnaporthe oryzae*) when expressed from the CaMV35S promoter, although rice fertility was seriously reduced (Ono et al., 2001). To see whether the mutated OsRac1(G19V) driven by endogenous promoter in the original locus would generate blast-fungus-resistant rice, the G56T nucleotide substitution was introduced in *OsRac1* through PNS-mediated GT and subsequent removal of the positive marker by Cre-*loxP* recombination (Dang et al., 2013). In the first step, *OsRac1-*GT occurred at a high frequency of 5.3% among the 94 calli surviving PNS; all five callus lines obtained carried the G56T substitution. Then, in the second step, β-estradiol-inducible *Cre* was transformed into each targeted callus line, and plants were regenerated from calli after induction of Cre expression. In total, seven fertile, *hpt*-free, rice plants with the G56T substitution in *OsRac1* were obtained from a single GT line. All plants expressed OsRac1(G19V) in the leaves with a blast fungus resistance phenotype; however, the level of the OsRac1(G19V) expression was unexpectedly low and the mutation was associated with a dwarf phenotype.

Excision of the selectable marker gene via the Cre-*loxP* or Flp-*FRT* system leaves recognition sequences for Cre and Flp recombinases—*loxP* and *FRT* sites, respectively—at the excised sites. For GT applications in the field, it is considered preferable to use marker excision systems that do not leave such sequences, to be more equivalent to spontaneous mutagenesis. The *piggyBac* transposon derived from the lepidopteran cabbage looper moth integrates into the host genome at TTAA elements and excises without leaving a footprint at the excised site (Cary et al., 1989). Using an assay system that allows transposition of *piggyBac* transposon to be visualized as luminescence derived from reconstituted luciferase expression cassettes, we demonstrated that the *piggyBac* transposon is capable of accurate and effective transposase-mediated transposition in plant cells (Nishizawa-Yokoi et al., 2014a).

**FIGURE 2:**
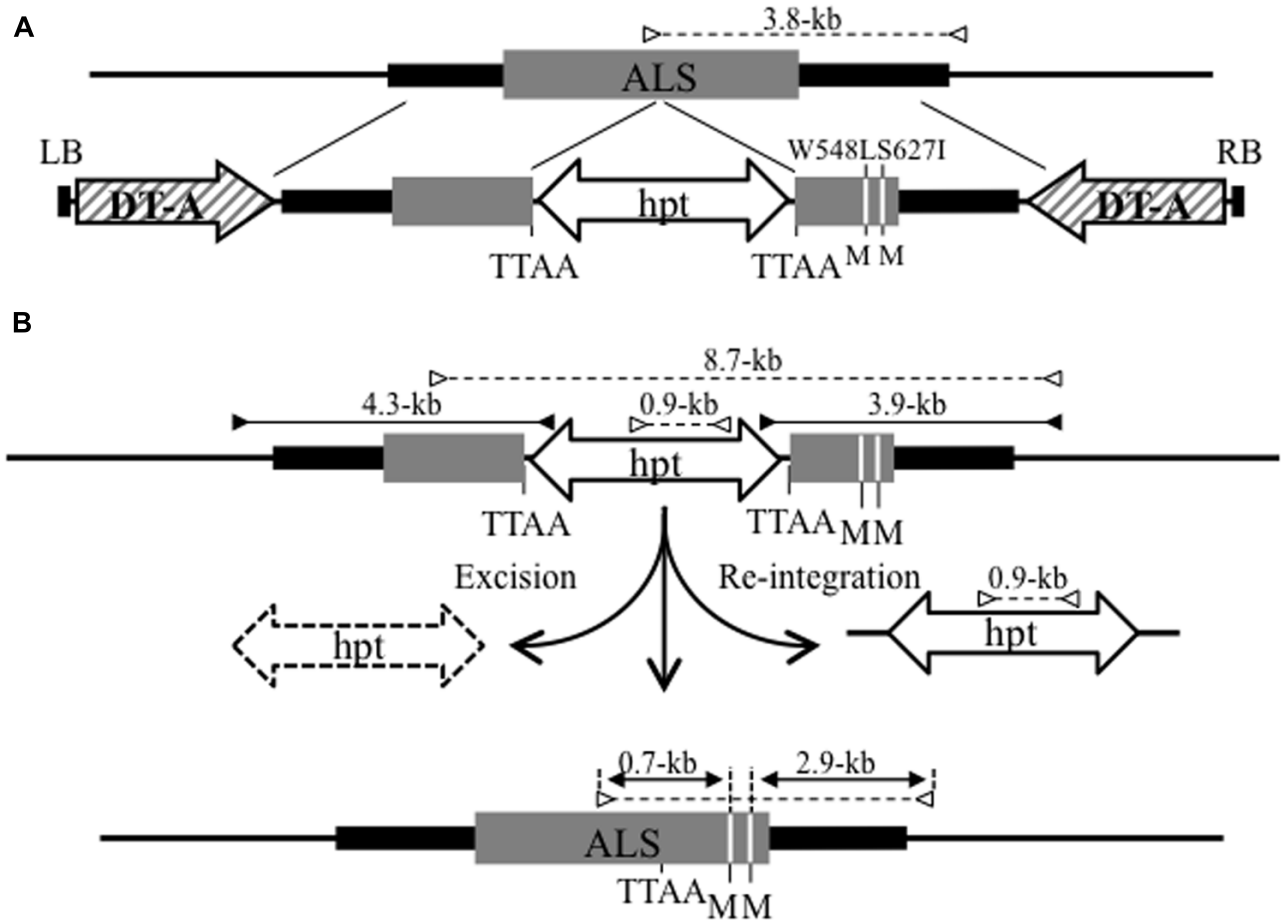
**Strategy for the introduction of point mutations into the *ALS* locus via GT and subsequent marker excision from the GT locus using the *piggyBac* transposon. (A)** Schematic diagram of GT at the *ALS* locus. The top line indicates the genomic structure of the wild-type *ALS* gene region. The bottom line shows the T-DNA region of the GT vector carrying *DT-A* as a negative selection marker and a 6.4-kb fragment containing an *ALS* coding region (gray box) with W548L and S627I mutations (white lines) and silent mutations (TTAA site added 301-bp upstream of W548L; GCTGAC to GAATTC) for the insertion of *piggyBac* transposon harboring *hpt* gene as a positive selection marker. The W548 L and S627I mutations create novel *Mfe* I restriction sites (M). LB, left border; RB, right border. **(B)** Strategy for precise marker excision from the GT locus using *piggyBac* transposon. The top line reveals the structure of the modified *ALS* locus resulting from HR between the GT vector and wild-type locus. The bottom line represents the *ALS* locus modified by GT and subsequent precise marker excision via *piggyBac* transposition. The primer sets used for PCR that identify transgenic calli in which a GT event occurred at the *ALS* locus are shown as black arrows. White arrows indicate the primer sets used for CAPS analysis to evaluate the frequency of marker excision via *piggyBac* transposition. The numbers on each arrow reveal the length of the PCR fragments.

To generate marker-free plants harboring only the desired mutation in the target locus, we attempted to introduce two point mutations accompanied by two amino acid changes—tryptophan (TGG) to leucine (TTG) at amino acid 548 (W548L), and serine (AGT) to isoleucine (ATT) at amino acid 627 (S627I)—into the rice acetolactate synthase (*ALS*) gene via PNS-mediated GT and subsequent marker excision by *piggyBac* transposition (**Figure 2**). Mutation of W548L/S627I in the *ALS* gene confers increased tolerance to the herbicide bispyribac (BS) on rice plants (Endo et al., 2007). Four-week-old rice calli were infected with *Agrobacterium* harboring a GT vector containing *hpt* and *DT-A* genes as PNS markers (**Figure 2A**). Four independent GT callus lines were identified by PCR analysis with the primer sets shown in **Figure 2B** and, among them, two lines were inoculated with *Agrobacterium* harboring a hyperactive *piggyBac* transposase (hyPBase, Yusa et al., 2011) expression vector driven by a constitutive promoter. Plants regenerated from hyPBase-expressing GT calli were subjected to marker excision analysis by cleaved amplified polymorphic sequence (CAPS) analysis. More than 90% of regenerated plants contained two point mutations in the *ALS* gene and lacked the *piggyBac* transposon carrying the *hpt* gene, suggesting that these regenerated plants indeed represented marker-free rice plants containing the desired mutations at the target locus (Nishizawa-Yokoi et al., 2014b). Our approach, i.e., GT with PNS and subsequent marker excision, provides a general strategy for targeted modification of endogenous genes in plants.

In a related genome editing strategy, DSBs were induced in the target gene using the zinc-finger nuclease (Shukla et al., 2009), TALENs (transcription activator-like effectors nuclease) from *Xanthomonas*, and CRISPR (clustered regularly interspaced short palindromic repeats)-associated (Cas9) systems in *Arabidopsis*, tobacco, maize, and rice (Zhang et al., 2013). DSBs are expected to enhance HR; indeed, effective HR induction was detected in an artificially targeted site (Puchta, 1999); however, for endogenous genes, most DSBs are repaired immediately by NHEJ and become associated with nucleotide deletions, substitutions, and insertions, resulting in gene-disruption-mediated mutants that could be screened for plant improvements (Shukla et al., 2009; Zhang et al., 2013; Puchta and Fauser, 2014). Precise nucleotide sequence design of a target gene by HR is still difficult even using induced DSB at a known target locus. Although PNS-mediated GT does not enhance HR, it can be combined with DSB induction in various plants with agricultural value, as well as in rice, in the search for new plant breeding technologies (NPBT).

## OUTLOOK

Positive–negative selection-mediated GT, which retains the unique competence for T-DNA mediated HR, has been developed in rice. In addition to gene knock-out, visualization of endogenous gene expression has been detected by gene knock-in. Further precise connection of a visual marker to the gene of interest will provide novel information about behavior of the protein in developing rice plants. Precise modification of target genes will be applicable to detailed functional analysis as well as rice breeding. Combination of PNS-mediated GT and genome editing strategy is expected to expand the availability of GT procedure and its application to various plants.

## Conflict of Interest Statement

The authors declare that the research was conducted in the absence of any commercial or financial relationships that could be construed as a potential conflict of interest.

## Abbreviations

DSBs: double strand break
*DT-A*: *diphtheria toxin A-fragment*
GT: gene targeting
*hpt*: *hygromycin phosphotransferase*
HR: homologous recombination
NHEJ: non-homologous end joining
PCR: polymerase chain reaction
PNS: positive–negative selection
T-DNA: transfer DNA
